# The relationship between physical activity and academic burnout among Yi primary school students in Southwest China: a moderated chain-mediation model

**DOI:** 10.3389/fpsyg.2026.1760564

**Published:** 2026-03-30

**Authors:** Wei Zhang, Xuping Zhao, Xiaoqian Hong, Yanxi Chen, Kang Zhang

**Affiliations:** School of Physical Education and Sports, Sichuan Normal University, Chengdu, China

**Keywords:** academic burnout, loneliness, mental toughness, physical activity, students

## Abstract

**Objective:**

This study examines the impact of physical activity on academic burnout among Yi elementary school students in Southwest China, as well as the mediating roles of psychological resilience and loneliness, and the moderating effect of grade level.

**Methods:**

A cross-sectional study was conducted with 1,104 Yi elementary school students (grades 5 ~ 6) in Liangshan Yi Autonomous Prefecture. Participants completed standardized measures of physical activity, mental toughness, loneliness, and academic burnout. Data were analyzed using correlation analysis and moderated chain mediation modeling with bootstrapping.

**Results:**

Physical activity is negatively correlated with loneliness and academic burnout, and positively correlated with mental toughness. Mediation analysis revealed three significant pathways: 1) the mediating effect of mental toughness alone (57% of the total effect), 2) the mediating effect of loneliness alone (26%), and 3) Chain-mediated effect between the two (17%). Grade level significantly moderated the final path from loneliness to academic burnout (*B* = 0.084, SE = 0.042, *p* < 0.05), with the effect being stronger for sixth graders.

**Conclusion:**

Physical activities alleviate academic burnout by reducing loneliness through enhanced mental toughness. The stronger association between loneliness and burnout among senior students underscores the necessity of implementing developmentally appropriate interventions.

## 1 Introduction

Academic burnout, a negative psychological state characterized by emotional exhaustion and disengagement from learning, poses a significant threat to student well-being and academic achievement globally (Tuominen-Soini and Salmela-Aro, 2014). In China, this issue is particularly acute, with studies indicating a high prevalence among primary school students (Ministry of Education of the People’s Republic of China, 2021; Li, 2024). The situation is even more challenging for ethnic minority students, such as the Yi community in Southwest China, who navigate the dual pressures of cultural integration and intense academic competition. These unique socio-cultural and educational challenges heighten their vulnerability to burnout, yet the specific psychological mechanisms at play within this population remain underexplored. Investigating these mechanisms is therefore critical for developing culturally sensitive interventions and promoting educational equity.

Physical activity (PA), defined as regular involvement in bodily movement aimed at enhancing physical and psychological functioning (Gan and Jiang, 2022). PA has emerged as a potent, non-stigmatizing intervention to counteract academic burnout. Physiologically, PA improves physical fitness (Fedewa and Ahn, 2011) and reduces the risk of chronic diseases (Janssen and LeBlanc, 2010). Psychologically, it can alleviate mental disorders (Poitras et al., 2016) and regulate emotional states (Zhou and Sun, 2025). Crucially, empirical evidence consistently demonstrates a negative correlation between PA and academic burnout (Chen et al., 2022; Lin et al., 2024; Tong and Jiang, 2024; Zhang et al., 2015; Yu et al., 2023). Grounded in theories such as Cultural Adaptation Theory, individuals may undergo cognitive, emotional, and behavioral adjustments when encountering different cultures. Yi elementary school students often need to reconcile differences between their ethnic culture and the mainstream culture within the school environment, potentially experiencing cultural adaptation stress that heightens the risk of academic burnout. If this process is accompanied by stress, it may adversely affect their academic engagement and psychological adjustment. PA may serve a unique cultural-psychological function in this process, fulfilling fundamental psychological needs for autonomy, competence, and relatedness, thereby replenishing the resources depleted by chronic academic stress. Accordingly, we propose:

*H1*: Physical activity will be negatively associated with academic burnout among Yi primary school students.

The cultural stress model further emphasizes that Yi students may encounter multiple stressors in educational settings, including language barriers, value conflicts, and a lack of belonging. If these stresses accumulate over time, they can easily lead to diminished learning motivation and emotional exhaustion (Ye et al., 2019). The protective effect of PA is unlikely to be direct; rather, it may operate through key psychological mechanisms. Among these, mental toughness is conceptualized as a psychological capacity progressively developed during cultural adaptation. It encompasses acceptance of cultural differences, integration of cultural identity under stress, and reconstruction of meaning in adversity, representing a critical personal resource (Fletcher and Sarkar, 2013; Punjani and Mevawala, 2019). According to the Conservation of Resources (COR) theory, individuals strive to obtain and protect resources, and stress occurs when these resources are threatened or lost. Studies indicate that mental toughness helps ameliorate persistent negative learning-related cognitions and facilitates adaptation (Gong et al., 2023). Physical activities have also been proven to boost elementary students’ self-confidence, thereby enhancing their mental toughness (Jiang Y. and Wang X., 2025). This enhanced resilience enables individuals to better utilize resources to cope with academic stress, thereby alleviating academic burnout (Fengjun et al., 2022). In summary, the psychological mechanisms of Yi students at the intersection of cultural identity and academic pressure are both unique and essential. Thus, we hypothesize:

*H2*: Mental toughness will mediate the relationship between physical activity and academic burnout.

Beyond intrapersonal resources, the social and emotional dimensions of school education are particularly crucial for Yi primary school students, who face significant educational challenges. Yi ethnic elementary students face unique adaptation challenges during cross-cultural schooling. Cultural cognitive differences and factors such as parents working away from home weaken traditional family support, making it easier for their emotional needs to be overlooked. This erodes psychological security and fosters enduring experiences of loneliness (Xu et al., 2025; Cao et al., 2022). From the perspective of cultural adaptation theory, their sense of loneliness stems not only from a lack of interpersonal connections but also from the internalization of cultural alienation (Kim, 2017). When individuals perceive their cultural identity as marginalized within a mainstream environment, they may experience a sense of “cultural loneliness.” This phenomenon is particularly pronounced when cultural adaptation fails to achieve integration or becomes entrenched in a state of marginalization. Numerous studies have also confirmed that the sense of loneliness experienced by students belonging to non-mainstream cultural groups stems not only from social factors but more significantly from cultural factors (Girmay and Singh, 2019; Franklin and Tranter, 2021). The role of PA in this process is complex: while they typically serve as structured platforms for peer interaction, their psychological effects are moderated by cultural contexts (Kim et al., 2020). If PA fail to accommodate cultural differences, they may instead accentuate the “otherness” of participants’ cultural identities, deepening their sense of alienation within the group. This provides a theoretical lens for interpreting the counterintuitive finding that “physical activity correlates positively with loneliness”: when PA fail to address cultural identity needs, participation itself may intensify cultural estrangement, elevating loneliness from a social dimension to a cultural one. Thus, we therefore propose:

*H3*: Loneliness will mediate the relationship between physical activity and academic burnout.

Crucially, we posit that mental toughness and loneliness do not operate in isolation but rather function as part of a sequential psychological cascade (Pervin, 1978). Grounded in the theory of individual-environment interaction, we propose a “resource empowerment” pathway: physical activity (PA) strengthens internal psychological resources, specifically mental toughness, which in turn enables individuals to more effectively engage with and adapt to their social environments. A student with higher mental toughness is more likely to interpret social challenges positively, exhibit greater social initiative, and persist in building relationships despite initial setbacks. This proactive engagement fosters the development of a richer peer network and more fulfilling interpersonal interactions, thereby mitigating feelings of loneliness (Papathanassoglou et al., 2015). This sequential process from intrapersonal resource to interpersonal outcome forms a chain of protection against academic burnout. Hence, we hypothesize that H4: Mental toughness and loneliness will sequentially mediate the relationship between physical activity and academic burnout (i.e., PA → mental toughness → loneliness → academic burnout). Thus, we therefore propose:

*H4*: Mental toughness and loneliness will sequentially mediate the relationship between physical activity and academic burnout.

Finally, we expect this complex interplay to be influenced by developmental stage, for which grade level serves as a proxy. As students transition from fifth to sixth grade, they face not only increasingly complex academic demands but also significant developments in cognitive and emotional regulation capabilities (Wang et al., 2021; Wigfield et al., 1996). According to developmental situationism theory, an individual’s development results from continuous interaction with an ever-changing environment. Against the backdrop of transitioning to middle school, students experience a sharp increase in their need for peer acceptance, social comparison, and identity formation, while the social environment exerts a more profound influence on their emotions and behavior. For sixth graders, their need for social belonging and cultural identity becomes more sensitive and intense (Verkuyten et al., 2019). If they persistently experience loneliness during this period, it may more directly and severely erode their engagement in learning and sense of meaning, thereby accelerating the pathway from loneliness to academic burnout. In contrast, fifth graders face relatively different developmental tasks and environmental demands, so loneliness may have a comparatively weaker impact on their academic outcomes. Therefore, we hypothesize that H5: Grade level will moderate the pathway between loneliness and academic burnout, with the relationship being stronger for sixth-grade students.

**Figure 1:**
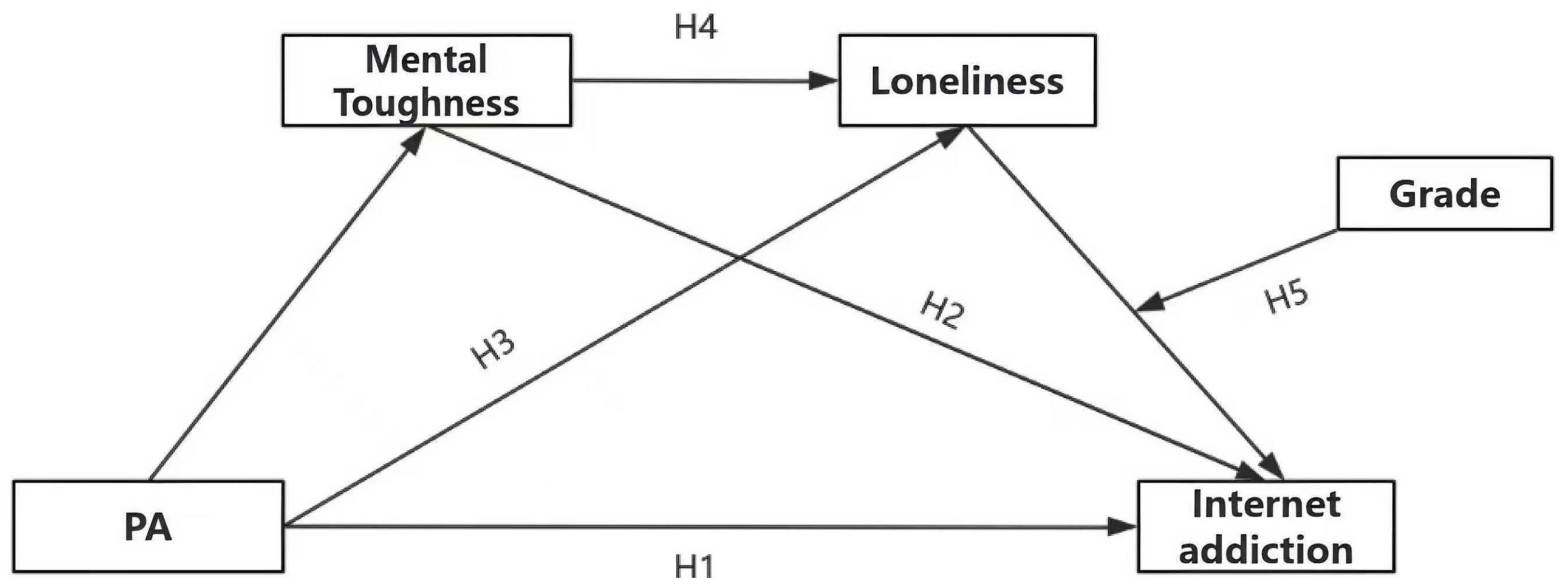
Hypothetical model diagram.

In summary, this study introduces for the first time a chained mediating pathway of “mental toughness—loneliness” among Yi ethnic students and examines the moderating role of grade level in this pathway. The findings provide empirical evidence for designing culturally sensitive, multidimensional intervention programs in primary and secondary schools within ethnic regions. This research offers valuable insights for enhancing students’ mental health in these areas and developing more inclusive school physical education and mental health education policies. However, compared to previous studies examining the relationship between physical activities and academic burnout among non-ethnic minority groups, this research exhibits gaps such as limited literature support. The hypothesized model is presented in Figure 1.

## 2 Research methodology

### 2.1 Participants

A cross-sectional study was conducted using a whole-cluster sampling method to recruit participants from four rural primary schools in Ningnan County, Liangshan Yi Autonomous Prefecture. All fifth and sixth-grade students from these schools were invited to participate. To calculate the required sample size, we conducted a pre-test power analysis using G*Power 3.1 software with *f*^2^ = 0.15, *α* = 0.05, and statistical power set at 0.80. The results indicated that the minimum required sample size is 129 participants. This study initially enrolled 1,115 students, far exceeding the threshold. The study received ethical approval from the Institutional Review Board of Sichuan Normal University (Approval No: 2023LSTY002). Written informed consent was obtained from all participants’ parents or guardians, and oral assent was obtained from each child prior to data collection. This survey was conducted by trained research assistants during physical education classes. After excluding 11 questionnaires due to incomplete responses or patterned answering, 1,104 valid questionnaires were retained (valid response rate = 99.0%). The final sample comprised 550 boys (49.9%) and 554 girls (50.1%), with 421 fifth graders (35%) and 683 sixth graders (65%). Although cluster sampling enhances efficiency, the research sample was drawn exclusively from four rural primary schools in Ningnan County, limiting its representativeness of Yi students from broader socioeconomic backgrounds. Detailed demographic characteristics are presented in Table 1.

**Table 1:** Basic information of subjects and the differences in academic burnout on demographic variables.

Group	Form	Reckoning	Percentage
Gender	Boy	550	49.9%
	Girl	554	50.1%
Grade	Grade 5	421	35%
	Grade 6	683	65%
Left-behind children	Yes	371	33.7%
	No	727	66.3%
An only child	Yes	112	10%
	No	990	90%
Family economic situation	Fine	121	11%
	General	888	80%
	Poorly	94	9%

Family financial status is based on individual subjective assessment.

### 2.2 Instruments

#### 2.2.1 Physical activity

This study employed the Physical Activity Rating Scale (PARS-3) (Jiayi et al., 2024), originally developed by Koshio Hashimoto and subsequently revised by Teh-ching Leung, to assess participants’ physical activity levels over the past month. The scale evaluates three core dimensions: intensity, duration, and frequency. Each dimension is scored on a 5-point Likert-type scale, with intensity and frequency rated from 1 to 5, and duration from 0 to 4. The total physical activity score was computed by multiplying the scores of the three dimensions: intensity × duration × frequency, ranging from 0 to 100. This scale has been validated among Chinese students. The present study confirms its good reliability and construct validity among Yi students in predictive contexts.

#### 2.2.2 Mental toughness

The Adolescent Mental Toughness Scale (RSCA) developed by Hu and Gan (Liu et al., 2020) was adopted. This 27-item instrument uses a 5-point Likert scale (1 = “not at all compliant” to 5 = “fully compliant”), including 12 reverse-scored items. Higher total scores indicate greater mental toughness. The scale comprises five factors: goal focus, emotional control, positive cognition, family support, and interpersonal assistance. The Cronbach’s alpha coefficient in this study was 0.746, with a KMO value of 0.855. Bartlett’s test of sphericity yielded a large chi-square value and was statistically significant (*p* < 0.001).

#### 2.2.3 Loneliness

The Child Loneliness Scale (CLS) was used to measure loneliness (Maes et al., 2017). This 24-item scale assesses four dimensions: loneliness, social competence evaluation, peer relationships, and satisfaction of needs for important relationships. It includes 8 filler items concerning personal preferences to help participants feel more at ease, and 16 core items (10 indicating loneliness, 6 indicating non-loneliness). Responses are recorded on a 5-point scale. The total score is derived from the core items, with higher scores indicating greater loneliness. Although the scale has been widely used internationally, it does not distinguish between social isolation and emotional isolation, potentially introducing latent cultural measurement biases. The Cronbach’s alpha coefficient in this study was 0.723, with a KMO value of 0.877. Bartlett’s test of sphericity yielded a large chi-square value and was statistically significant (*p* < 0.001).

#### 2.2.4 Academic burnout

The Adolescent Academic Burnout Scale (ASBI) developed by Li et al. (2025) was used. This self-report scale comprises 16 items across three dimensions: physical and mental exhaustion (4 items), academic detachment (5 items), and reduced personal accomplishment (7 items). Higher total scores indicate more severe academic burnout. The Cronbach’s alpha coefficient in this study was 0.706, with a KMO value of 0.852. Bartlett’s test of sphericity yielded a large chi-square value and was statistically significant (*p* < 0.001).

### 2.3 Data analysis

Data were analyzed using SPSS 27.0 and the PROCESS 4.2 macro. The analytical procedures included tests for common method bias, descriptive statistics, correlation analyzes, and regression analyzes. To examine the chained mediation model, the PROCESS macro with Model 6 was employed. To precisely test whether the moderating variable affects only one link in the mediating pathway, use Model 87. The study controlled for variables including gender, left-behind children, and only children. Bias-corrected and accelerated (BCa) bootstrap confidence intervals based on 5,000 resamples were used to estimate indirect effects, enhancing the robustness and accuracy of the inferential results.

## 3 Results

### 3.1 Common method bias test

Harman’s single-factor test was conducted to assess common method bias due to the use of self-report measures. Exploratory factor analysis revealed 16 factors with eigenvalues greater than 1. The largest factor accounted for 13.23% of the variance, below the 40% threshold, indicating that common method bias was not a significant concern. Although common method bias has been detected, self-reported data may still exhibit potential for common method variance inflation.

### 3.2 Descriptive statistics and correlations

Descriptive statistics and correlations are presented in Table 2. As shown in the figure, physical activity exhibits a significant negative correlation with academic burnout and loneliness, and a significant positive correlation with mental toughness. Mental toughness is negatively correlated with loneliness. Academic burnout shows a significant negative correlation with mental toughness and a positive correlation with loneliness.

**Table 2:** Descriptive statistics and correlations among study variables.

Variant	M	SD	1	2	3	4	5	6	7	8	9
1. Gender			1								
2. Grade			0.034	1							
3. Left-behind children			0.060*	0.002	1						
4. An only child			0.036	−0.004	0.119^**^	1					
5. Family economic situation			−0.072^*^	0.140^**^	−0.087^**^	0.029	1				
6. PA	14.91	15.42	−0.240^**^	−0.002	0.016	0.006	0.002	1			
7. Academic burnout	41.70	8.82	0.011	0.078*	−0.009	−0.022	0.056	−0.076^*^	1		
8. Mental toughness	89.16	13.67	−0.069^*^	−0.045	0.060^*^	0.089^**^	−0.038	0.114^**^	−0.554^**^	1	
9. Loneliness	56.21	9.13	−0.001	−0.036	−0.018	0.017	0.060*	−0.162^**^	0.496^**^	−0.515^**^	1

^**^为*p*<0.01, ^***^p<0.001. The numerical codes above the table correspond to the variable names following the numbers on the left.

### 3.3 Mediating analysis

**Figure 2:**
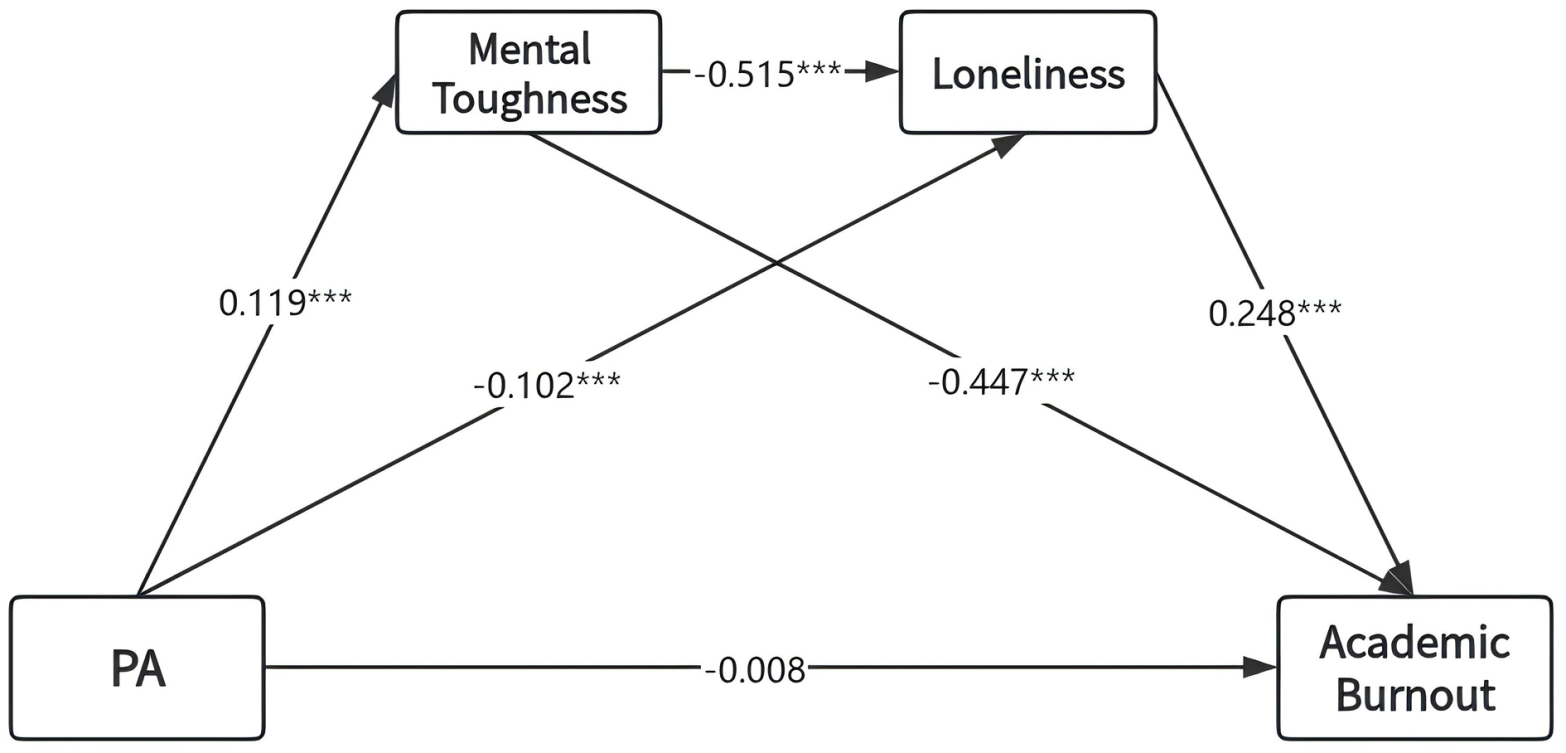
Mediating model of mental toughness and loneliness between PA and academic burnout.

We tested a mediation model using PROCESS Model 6 (5,000 bootstrap samples), controlling for gender, left-behind status, and only-child status. Physical activity was the independent variable, academic burnout the dependent variable, and mental toughness and loneliness were mediators. As shown in Table 3 and Figure 2, physical activity negatively predicted academic burnout (*β* = −0.102, *p* < 0.01) and loneliness (*β* = −0.102, *p* < 0.001). When the mediating variable was included, physical activity had a positive predictive effect on mental toughness (*β* = 0.119, *p* < 0.001) mental toughness negatively predicted loneliness (*β* = −0.515, *p* < 0.001) and academic burnout (*β* = −0.447, *p* < 0.001), while loneliness (*β* = 0.248, *p* < 0.001) positively predicted academic burnout. These results support Hypothesis 1.

**Table 3:** Results of the mediation effect analysis.

Predictor variable	Academic burnout			Mental toughness			Loneliness			Academic burnout		
	*β*	*SE*	*t*	*β*	*SE*	*t*	*β*	*SE*	*t*	*β*	*SE*	*t*
Constant	41.933	2.380	17.616^***^	83.805	3.656	22.921^***^	69.848	2.886	24.198^***^	58.249	2.857	20.387^***^
Gender	−0.016	0.553	−0.515	−0.050	0.851	−1.600	−0.071	0.552	−2.648^**^	−0.027	0.443	−1.087
Left-behind children	0.007	0.568	0.241	0.048	0.873	1.582	0.020	0.567	0.778	0.030	0.452	1.230
An only child	−0.027	0.887	−0.905	0.074	1.362	2.471	0.048	0.886	1.857	0.003	0.708	0.138
Family economic situation	0.059	0.604	1.938	−0.046	0.928	−1.532	0.041	0.602	1.585	0.022	0.481	0.917
PA	−0.102	0.018	−3.251^**^	0.119	0.028	3.824^***^	−0.102	0.018	−3.793^***^	−0.008	0.014	−0.330
Mental toughness							−0.515	0.020	−19.783^***^	−0.447	0.018	−15.795^***^
Loneliness										0.248	0.024	8.749^***^
*R*	0.102			0.167			0.531			0.613		
*R^2^*	0.010			0.028			0.282			0.375		
*F*	2.841			7.793			85.400			108.821		

Bootstrap analysis (Table 4) confirmed the significance of the indirect effects. The specific indirect effect through mental toughness alone was −0.030 [95% CI (−0.049, −0.013)], accounting for 57% of the total effect. The indirect effect through loneliness alone was −0.014 [95% CI (−0.024, −0.006)], accounting for 26%. The serial indirect effect through loneliness then mental toughness was −0.009 [95% CI (−0.015, −0.004)], accounting for 17%. As all confidence intervals excluded zero, Hypotheses 2, 3, and 4 were supported.

**Table 4:** Bootstrap analysis of the mediation effects between physical activity and academic burnout.

Modeling effect	Effect	Boot SE	Bootstrap 95% CI		Percentage
			LLCI	UICI	
Aggregate effect	−0.058	0.018	−0.093	−0.023	/
Direct effect	−0.005	0.014	−0.033	0.023	0%
Total indirect effect	−0.053	0.013	−0.080	−0.029	100%
Physical activity →Mental toughness →Academic burnout	−0.030	0.009	−0.049	−0.013	57%
Physical activity →Loneliness →Academic burnout	−0.014	0.005	−0.024	−0.006	26%
Physical activity → Loneliness → Mental toughness → Academic burnout	−0.009	0.003	−0.015	−0.004	17%

### 3.4 Moderating effects test

**Figure 3:**
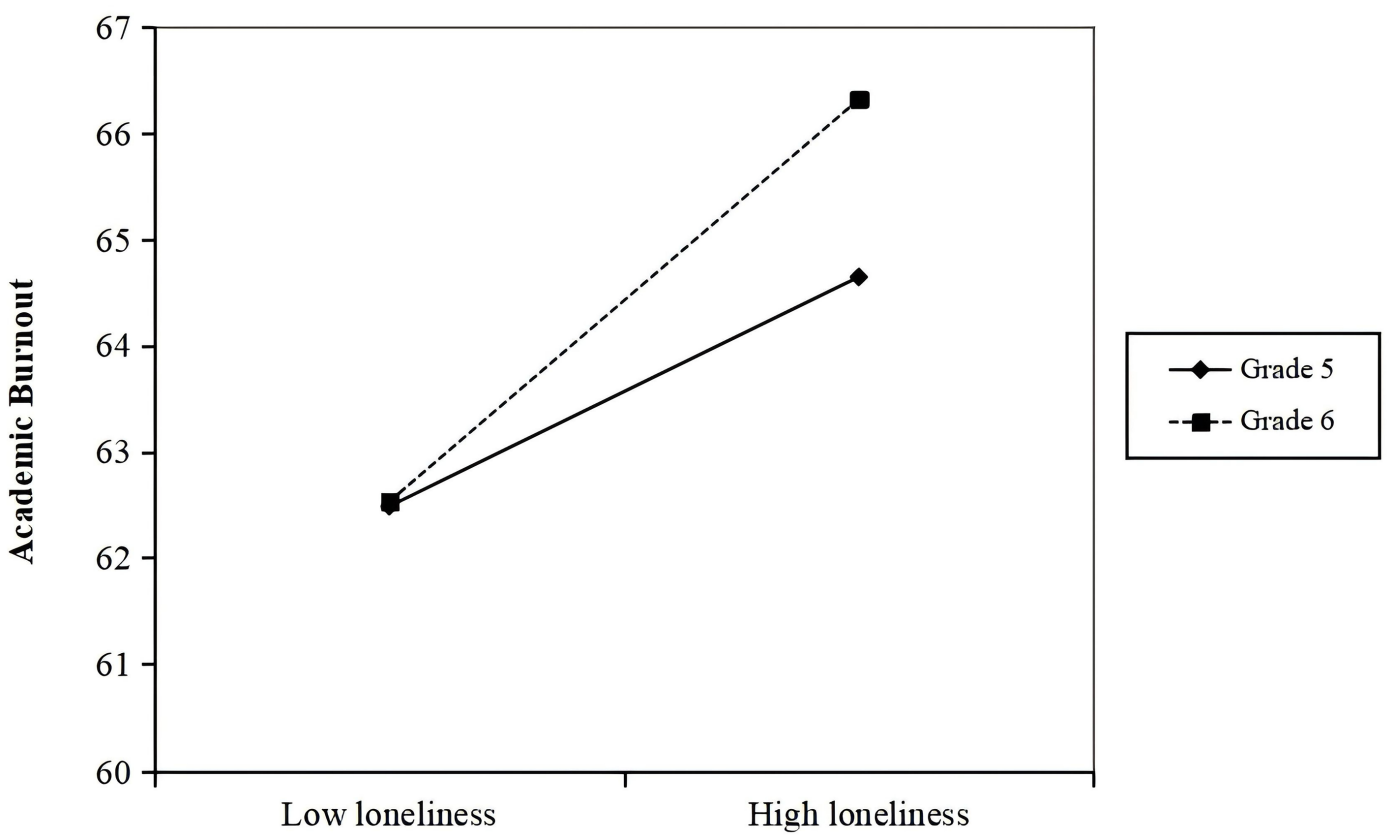
The moderating role of grade in the relationship between loneliness and academic burnout.

**Figure 4:**
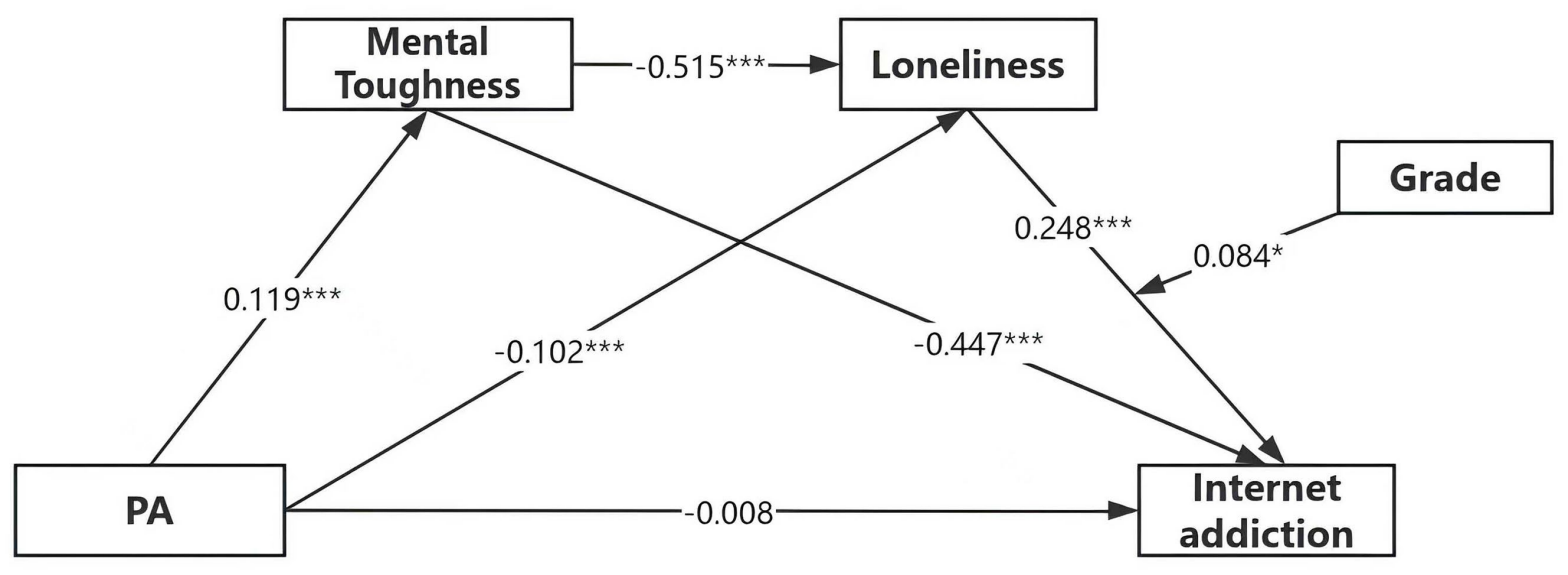
Moderated chain-mediated model with grade as a moderator.

To test the moderating role of grade (Hypothesis 5), we used PROCESS Model 87. Results (Table 5) indicated a significant interaction between loneliness and grade level on academic burnout (*B* = 0.084, *p* < 0.05). Simple slope analysis (Figure 3) revealed that the negative relationship between loneliness and academic burnout was significant for both fifth graders (*B* = 0.165, *p* < 0.001) and sixth graders (*B* = 0.250, *p* < 0.001), but significantly stronger for sixth graders. Thus, Hypothesis 5 was supported (Figure 4).

**Table 5:** Regression results testing the moderating role of grade level.

Variant	Mental toughness			Loneliness			Academic burnout		
	*B*	*SE*	*t*	*B*	*SE*	*t*	*B*	*SE*	*t*
Constant	87.430	0.567	154.097^***^	72.708	1.744	41.682^***^	62.318	3.470	17.961^***^
Gender	−1.361	0.850	−1.601	−1.462	0.552	−2.648^**^	−0.480	0.443	−1.086
Left-behind children	1.382	0.873	1.582	−0.441	0.567	0.778	0.501	0.453	1.108
An only child	3.366	1.362	2.471^*^	1.645	0.886	1.857	0.066	0.707	0.094
Family economic situation	−1.421	0.928	−1.532	0.954	0.602	1.585	0.270	0.486	0.557
PA	0.116	0.026	4.379^***^	−0.055	0.017	−3.199^***^	0.004	0.014	0.030
Mental toughness				−0.3383	0.019	−19.637^***^	−0.283	0.018	−15.745^***^
Loneliness							0.081	0.072	1.130
Grade							−2.257	1.636	−1.379
Int_1							0.084	0.042	2.003^*^
*R^2^*	0.017			0.275			0.380		
*F*	19.180^***^			209.714^***^			134.760^***^		

## 4 Discussion

### 4.1 Physical activity as a protective factor against academic burnout

Our study provides robust evidence that physical activity serves as a significant negative predictor of academic burnout among Yi elementary school students, thereby confirming our hypothesis 1. As previous research has demonstrated, prevention frameworks incorporating exercise-based mental health interventions have emerged as promising non-pharmacological strategies for reducing suicide risk and enhancing existing prevention models (Kanani et al., 2025). This finding aligns with the Conservation of Resources theory, which posits that individuals strive to build and protect their resources. Chronic academic pressure depletes students’ mental resources, leading to burnout. We contend that physical activity functions as a key resource-gaining strategy. This finding aligns with the research by Jiang Y. and Wang T. (2025), which indicates that after-school extended physical education services exert a negative influence on academic burnout. It also supports Deng et al. (2025) conclusion that academic burnout and physical exercise exhibit a negative correlation. Academic burnout not only hinders academic achievement but also exerts long-term negative effects on an individual’s mental health and social adaptation (Chung and Park, 2024), it often manifests as diminished self-worth, estranged peer relationships, and avoidance of learning activities (Chen et al., 2023; Ni et al., 2025). Through the mechanism of self-determination theory, PA likely fulfills basic psychological needs for skill mastery, voluntary movement, social interaction, thereby replenishing depleted resources that protects against the development of burnout (Ng, 2023; Vasconcellos et al., 2020; Chen et al., 2024). This perspective moves beyond a simple correlational finding and positions PA as an active, resource-based intervention in a high-demand educational context.

### 4.2 The mediating role of mental toughness

Supporting Hypothesis 2, our results identified mental toughness as a critical mediating mechanism. This finding can be powerfully interpreted through the lens of COR theory, where mental toughness is conceptualized as a vital personal resource bank. Physical activity, particularly in the form of structured and challenging exercises, acts as a “resource passageway.” It not only builds physical fitness but also trains psychological adaptability and stress tolerance. Zhang et al. (2025) indicate that physical exercise exhibits a significant positive correlation with mental toughness. Neurobiologically, this process may be facilitated by exercise-induced increases in Brain-Derived Neurotrophic Factor (BDNF), which supports neural plasticity and stress resilience (Peng et al., 2025). Additionally, the findings of this study support a negative association between mental toughness and academic burnout. From an expanded perspective of resource conservation theory, mental toughness can be viewed as a positive trait that facilitates the active conservation and acquisition of resources (Hobfoll, 2011; Yu and Chae, 2020). Therefore, students with higher levels of mental toughness tend to exhibit more effective coping strategies for academic stress and more positive challenge assessment patterns, resulting in lower levels of core burnout symptoms in their academic experiences (Firman et al., 2025). In summary, students with enriched mental toughness resources may be better equipped to navigate academic setbacks, reframe challenges, and prevent resource loss, thereby directly mitigating the core components of academic burnout.

### 4.3 The mediating role of loneliness

Further validation of Hypothesis 3 confirms loneliness as a significant mediating variable. This finding underscores the social dimension of resource conservation. Loneliness is fundamentally a severe deprivation of social resources. According to the social support buffer model (Cohen and Wills, 1985), when these resources are lacking, academic stress directly and comprehensively impacts individuals. This finding is consistent with Bin L’s (Chen et al., 2025) research, which demonstrated that loneliness mediates the relationship between PA and academic burnout. For Yi students, the emotional energy required to cope with loneliness and academic pressure accelerates resource depletion. Frequent interactions foster friendship formation and trust development, fulfilling their need for belonging and effectively alleviating loneliness stemming from social deprivation. Therefore, most studies indicate that regular exercise helps alleviate feelings of loneliness (Liang et al., 2025; Padial-Ruz et al., 2020). In environments lacking cultural support, perceived discrimination intensifies feelings of loneliness, further highlighting the role of socially inclusive sports activities in mitigating such effects (Salway et al., 2020; Page et al., 1992). Therefore, physical activity can serve not only as a means to alleviate loneliness but also as an active mechanism for maintaining psychological resources, thereby offering a potential pathway to mitigate academic burnout.

### 4.4 The sequential mediation pathway: from mental toughness to loneliness

Finally, the chain-mediated effect of mental toughness and loneliness between PA and academic burnout, Hypothesis 4 was confirmed. This finding supports the cascading protective model of “physiological arousal—psychological empowerment—social connection,” offering new insights into the underlying mechanisms through which PA alleviates academic burnout. The chained mediation model identified in this study reveals an ideal dynamic process: Physical activity enhances mental toughness via neurobiological adaptation and behavioral training (Arida and Teixeira-Machado, 2021). Enhanced mental toughness enables individuals to interpret interpersonal difficulties more positively and increase social engagement, thereby significantly reducing feelings of loneliness (Gross, 1998). Alleviating loneliness further frees up psychological resources otherwise consumed by emotional exhaustion, enabling elementary students to sustain their engagement in learning and ultimately alleviate academic burnout (Singh et al., 2021).

### 4.5 The moderating influence of grade level

Additionally, grade level moderates the relationship between loneliness and academic burnout, supporting Hypothesis 5. As grade level increases, the interaction between academic stressors and self-perception undergoes changes (Cantin and Boivin, 2004). The stronger negative association between loneliness and academic burnout for sixth graders can be explained by Stage-Environment Fit Theory (Eccles, 2004). Entering sixth grade signifies a significant increase in academic demands and expectations, leading to more complex mechanisms in the formation of learning fatigue (Graves, 2018). In this high-risk context, the nature of loneliness may undergo transformation. For sixth graders, social isolation is not merely an emotional distress but a direct threat to academic survival. Peer support becomes crucial for navigating complex curricula and pressures. Consequently, loneliness among sixth graders exhibits a stronger negative predictive effect on academic burnout. This indicates that interventions addressing academic burnout among Yi elementary students must fully account for the developmental characteristics of this grade level to formulate more targeted support strategies.

Research Limitations and Future Directions: First, the use of cross-sectional data limits the ability to establish causal inferences among the variables, and did not control for school-level variables. Future studies should employ longitudinal or experimental designs and utilize multilevel modeling (MLM) to more rigorously examine the temporal and causal relationships underlying the observed associations. Second, future research should incorporate these variables to more comprehensively reveal cultural psychological mechanisms. Qualitative research may also be conducted to supplement the findings of quantitative research. Finally, this study constructed only a mediation model with moderation. Future research could explore effective pathways for integrating ethnic traditional sports into curricula by developing more complex integrated models and conducting randomized controlled trials, thereby providing empirical evidence for formulating culturally responsive educational policies.

## 5 Conclusion

This study demonstrates that physical activity significantly alleviates academic burnout among Yi elementary school students by reducing loneliness levels through enhanced mental toughness. The stronger linkage between loneliness and burnout among sixth graders further highlights the developmental sensitivity of this process. It provides empirical evidence for designing physical activity interventions within school education that are both developmentally sensitive and culturally responsive.

## Data Availability

The original contributions presented in the study are included in the article/supplementary material, further inquiries can be directed to the corresponding author.
